# Network analysis of the social and demographic influences on name choice within the UK (1838-2016)

**DOI:** 10.1371/journal.pone.0205759

**Published:** 2018-10-31

**Authors:** Stephen J. Bush, Anna Powell-Smith, Tom C. Freeman

**Affiliations:** 1 The Roslin Institute, University of Edinburgh, Midlothian, United Kingdom; 2 Flourish, Kiln Enterprises Ltd, London, United Kingdom; Indiana University, UNITED STATES

## Abstract

Chosen names reflect changes in societal values, personal tastes and cultural diversity. Vogues in name usage can be easily shown on a case by case basis, by plotting the rise and fall in their popularity over time. However, individual name choices are not made in isolation and trends in naming are better understood as group-level phenomena. Here we use network analysis to examine onomastic (name) datasets in order to explore the influences on name choices within the UK over the last 170 years. Using a large representative sample of approximately 22 million forenames from England and Wales given between 1838 and 2014, along with a complete population sample of births registered between 1996 and 2016, we demonstrate how trends in name usage can be visualised as network graphs. By exploring the structure of these graphs various patterns of name use become apparent, a consequence of external social forces, such as migration, operating in concert with internal mechanisms of change. In general, we show that the topology of network graphs can reveal naming vogues, and that naming vogues in part reflect social and demographic changes. Many name choices are consistent with a self-correcting feedback loop, whereby rarer names become common because there are virtues perceived in their rarity, yet with these perceived virtues lost upon increasing commonality. Towards the present day, we can speculate that the comparatively greater range of media, freedom of movement, and ability to maintain globally-distributed social networks increases the number of possible names, but also ensures they may more quickly be perceived as commonplace. Consequently, contemporary naming vogues are relatively short-lived with many name choices appearing a balance struck between recognisability and rarity. The data are available in multiple forms including via an easy-to-use web interface at http://demos.flourish.studio/namehistory.

## Introduction

Choosing the name of a newborn is a dilemma faced by all parents. Each name carries connotations–personal, societal, cultural and religious–and may be considered a symbolic expression of parental expectation, a statement of individuality and/or of group belonging. As a representation of self-identity [1], a name acts as a template for the development for self-image, indicating the child’s position in status hierarchies of gender, race, and social class, thereby influencing the behaviour of others towards them [2]. Names act as identity stereotypes [3] and affect perceptions of moral character [4], professional competence [5], educational ability [6,7], and physical attractiveness [8].

Whatever the reasoning behind the choice of a name, it is always made within the context of a time and place–and as the perception of a name changes over time, so does its popularity. This can influence parental choice and result in naming fads, sudden and short-lived increases in popularity, and vogues, a longer term gain or loss of popularity. For instance, some names gain rapid popularity through positive association with high-achieving or famous individuals, resulting in a fad. Conversely, should a name acquire negative association by the actions of certain bearers, such as tyrants [9], they may subsequently be avoided. By popularising famous individuals, television and film, amongst other media, associate a large pool of names with particular characteristics, creating and maintaining culturally-determined stereotypes [10]. As a consequence of their allusion to a stereotypical identity, names may then be selected, often unconsciously, transferring parental predilections to their children [11].

By contrast, naming vogues can reflect more complex, longer-term changes to a population, both cultural and demographic, that alter the perception of a name and affect its popularity over time. Although a precise distinction between fads and vogues is difficult to define, both are easily visualised on a case by case basis as a line graph. Whilst this provides a simple and clear representation of individual name usage over time, it is of limited use in understanding naming trends at the population level: names do not exist in isolation, and naming trends over many years are better understood as a group-level phenomenon. Taste is a continually changing collective behaviour, affected both by external social forces and internal mechanisms of change [12]. As a cultural trait, names are of particular interest for studying the internal drivers of cultural evolution as their popularity depends entirely on cultural influence–names are essentially unconstrained individual choices, shorn of commercial interest [13]. Consequently, numerous models have been proposed to explain the volatile dynamics of name usage, as this can highlight mechanisms of cultural change [14–17].

This study set out to examine influences on name choice in the UK. To this end, we have employed network analysis, a practical application of graph theory widely used to analyse data in many academic disciplines, including sociology, biology, computer science, and physics [18], and increasingly in onomastics (the study of the history and origin of proper names), where–for instance–networks of forename-surname pairs have revealed the ethnic sub-structure of whole populations [19]. Here, we consider a set of names to be the elementary components (nodes) of a network. In this context edges (relationships between names) are based on the Pearson correlation measure, as calculated by comparing the usage profile between one name and another. By comparing the popularity of each name over time with every other name, a distance matrix is generated, whereby the closer usage profiles are, the higher the correlation value. Use of a correlation threshold means that a name is only connected to other names that show a similar trend. A network graph can be used to visualise these relationships. By visualising the usage of names in this manner, trends in the use of any given name can be analysed alongside the relationships between them. In this respect, common factors may be found to underlie the popularity of particular groups of names at particular times, factors that may not be considered if names were analysed independently.

As a primary source of data, we mined a series of local birth registers. By so doing we obtained a large-scale sample of first and middle name information for approximately 22 million individuals born in England and Wales between 1838 and 2014. Using this dataset, along with a dataset from the UK Office for National Statistics (ONS) containing a complete population sample of births registered between 1996 and 2016, this study demonstrates how network graphs can condense complex onomastic data into an accessible, and visually intuitive, format. To illustrate the utility of network analysis for onomastic analysis, we examine the topographical structure of this network of name choices, isolating subsets of names whose popularity shows vogue-like behaviour over time. As well as relating name usage to historic events, such as known waves of migration, we find that many vogues in name usage likely reflect an individual’s perception of a given name. Other patterns of name use reflect societal changes to the UK, particularly among contemporary naming trends, in which there has been a significant increase in diversity over the last few decades. In general, these data expose many interesting associations between names and historic events, as well as societal changes that lead to departure from former naming traditions, and demographic changes broadening the ethnic and cultural composition of the UK.

## Materials and methods

### Primary data

A corpus of names was obtained from the UK ‘local BMD’ project (http://www.ukbmd.org.uk/local), an ongoing volunteer effort to transcribe the local indices of the UK births, marriages and deaths (BMD) registers for digital preservation. BMD registration began in England and Wales in 1837, and became compulsory with the Births and Deaths Registration Act 1875. Each quarter, copies of the BMD indices generated locally are sent to the General Register Office in London, where they are re-transcribed to form a national catalogue. However, the data is not publicly available in a form amenable to large-scale analysis, the websites hosting the records only permitting the bulk download of 25 years’ worth of records at a time for a single letter, i.e. a subset of records with surnames beginning with A, and so on. To obtain the dataset used here, 1716 files spanning all years and regions had to be individually downloaded.

Data was collated from all participating areas in the UK local BMD project: the cities, counties and regions of Bath, Berkshire, Cheshire, Cumbria, Lancashire, North Wales, Staffordshire, West Midlands, Wiltshire, and Yorkshire (Table 1), and downloaded on 12^th^ September 2016. Each of these areas constitutes a different record transcription project. These are run by volunteers, with larger volunteer efforts in different areas. As such, the data is non-uniform both in terms of records per geographical region and depth of coverage over time. Several of these projects (Berkshire, Cumbria, North Wales) are not actively maintained, and contain no new birth records for 4–5 years prior to data collation. The available fields for each birth record were the first name, middle name(s) and surname, year of birth, district in which the birth was registered, and identification number. The data includes 143,259 unique names from approx. 22 million individuals over 177 years, from 1838 (the first complete year of BMD registration) to 2014. This approximates 130,000 to 230,000 records per year from 1838–1950, 25,000 to 100,000 records per year from 1951–2000, and 5000 to 15,000 records per year from 2001 to 2014. As such, we assume its scope is sufficiently broad to be representative of UK naming patterns.

**Table 1:** Local birth registers from which a corpus of names from England and Wales was obtained.

Region	URL	Date records were last updated	No. of usable birth records	Years covered
Bath	http://www.bathbmd.org.uk/	21st July 2016	485,315	1838–2007
Berkshire	http://www.berkshirebmd.org.uk/	27th Oct 2011	282,195	1838–1968
Cheshire	http://www.cheshirebmd.org.uk/	1st Sept 2016	3,325,710	1838–2014
Cumbria	http://www.cumbriabmd.org.uk/	12th Dec 2011	277,482	1838–2009
Lancashire	http://www.lancashirebmd.org.uk/	9th Sept 2016	9,885,291	1838–2000
North Wales	http://www.northwalesbmd.org.uk/	22nd July 2012	1,336,027	1838–1995
Staffordshire	http://www.staffordshirebmd.org.uk/	9th Sept 2016	1,880,777	1838–2008
West Midlands	http://www.westmidlandsbmd.org.uk/	3rd June 2016	1,414,097	1838–2002
Wiltshire	http://www.wiltshirebmd.org.uk/	1st Sept 2016	263,248	1838–1948
Yorkshire	http://www.yorkshirebmd.org.uk/	8th Sept 2016	3,487,143	1838–2010
		Total	22,637,285	

### Data cleansing

For this analysis, typographical errors were manually corrected if the name as transcribed was unpronounceable (for instance, Wlliam instead of William), or if there was an unambiguous character inversion (Geroge instead of George) or duplication (Aaaron instead of Aaron). Names were unaltered if they could plausibly be considered as a spelling variant, for instance, Barbera (a red wine grape; 7 records) as a variant of Barbara (approx. 60,000 records). Uninformative entries were excluded such as names registered as a single initial, or with generic placeholders (such as Boy or Girl [which together account for approx. 75,000 records], Son, Daughter, Foundling, Un-named and Deceased), as were unrecognisably abbreviated names. Conventionally accepted abbreviations, such as Edw’d for Edward, and Wm for William, were included. The subjectivity of these corrections is acknowledged. Typographical corrections made to the data, and those entries not considered names, are given in S1 and S2 Tables, respectively. Finally, names that were not present in the dataset as a whole >50 times were removed. Name frequency was recorded as the percentage of total names registered in a given year. In total, the cleaned dataset contains names from 22,637,285 individuals.

The UK local BMD records do not list a person’s assigned sex. For the purposes of this study, the gender associated with a name was inferred by reference to census data from the United States (as in [20])–comparing names to a corpus of first names collated by the United States Social Security Administration in the period 1880 to 2015 (https://www.ssa.gov/OACT/babynames/names.zip; accessed 15^th^ November 2016). The gender of a name was assigned as either male or female if it was associated with a single gender in >95% of cases (this dataset acknowledges only two genders). However, numerous names are unisex making gender assignment ambiguous. For example, without contextual information Nicola may be an English female name or an Italian male name. Although unisex names, such as Leslie and Robin, are more likely to be female [21], we have not sought to assign them in this corpus. In total the dataset includes 3,246 names: 1,656 female, 1221 male, and 260 unisex. For 107 names we were unable to assign a gender.

### Contemporary name usage data

The local BMD dataset has comparatively low coverage of contemporary birth records. To supplement this data, complete records of all live births in England and Wales from 1996 to 2016 were obtained from the UK Office for National Statistics (ONS) (https://www.ons.gov.uk/file?uri=/peoplepopulationandcommunity/birthsdeathsandmarriages/livebirths/datasets/babynamesenglandandwalesbabynamesstatisticsgirls/2016/adhocallbabynames1996to2016.xls, accessed 1^st^ February 2018). Compared to the local BMD dataset, this is deeper in coverage but narrower in scope, containing 12,985,140 records (approximately 600,000 per year) and representing 34,202 unique forenames (to protect the identity of individuals, neither middle names nor forenames registered to < 3 births per year are included). Individual name usage profiles for the ONS dataset have previously been made available online (http://names.darkgreener.com).

### Network analysis

Network analysis was performed using Graphia Professional (Kajeka Ltd, Edinburgh, UK), previously called BioLayout *Express*^3D^, a tool originally designed to analyse gene expression data [22,23]. Upon loading the data into the tool, expressed as a percentage of total registered names per year (the ‘usage profile’ of each name), a Pearson correlation matrix is calculated. This compares the profile of an individual name’s use over the years with every other name’s use, expressing the results between -1 (anti-correlated) and +1 (perfectly correlated). A correlation threshold is then applied removing weak correlations; in the case of the BMD dataset, correlations where *r* < 0.6. A network graph was constructed by connecting nodes (names) with edges (correlations exceeding the threshold). This threshold was determined empirically such that the resultant graph included the majority of names connected by a minimum of edges, thereby revealing the data’s structure. The threshold is significantly higher than correlations that would be expected by chance, thereby minimising spurious associations. This graph was then subjected to cluster analysis using the Markov clustering algorithm (MCL) [24] with an inflation value (which determines cluster granularity) of 3. This identifies groups of names (clusters) which have similar usage profiles. Clusters are numbered in descending order of size.

### Data usage statement

The website hosting the UK local BMD project (http://www.ukbmd.org.uk) is operated by Weston Technologies Limited (Crewe, Cheshire, UK). This company is the owner or license-holder of the intellectual property constituting the birth records–obtained from the subsidiary websites in Table 1 –as detailed at https://www.ukbmd.org.uk/TermsAndConditions (accessed 12^th^ September 2016). Under section 29A of the UK Copyright, Designs and Patents Act 1988, a copyright exception permits copies to be made of lawfully accessible material in order to conduct text and data mining for non-commercial research. This exception is invoked here.

### Data availability

The BMD corpus of names is presented in various forms: as a rank order of names (in both the first and middle position) by number of registered births per year (S3 and S4 Tables, respectively), and by the total number of births across all years sampled (S5 Table). An overview of the data is also provided, as a table of summary statistics: the number of usable records registered per year, the number of unique names per year, most popular forename and middle name per year, and measures of forename diversity and the surname-to-forename usage ratio (an indicator of which forenames are more likely to be transferred uses of surnames) (S6 Table). For each forename, frequently co-occurring middle names (those with > 100 records) are listed in S7 Table. Usage profiles are available as absolute numbers and proportions both for forenames (1,656 female, 1,221 male, 367 unisex/unknown) and middle names (820 female, 849 male, 1977 unisex/unknown) (S8–S11 Tables). Rare names (< 50 total records across all years) were excluded from analysis.

These tables are extensive but not exhaustive and do not exclude the possibility that errors remain in the corpus.

**Fig 1:**
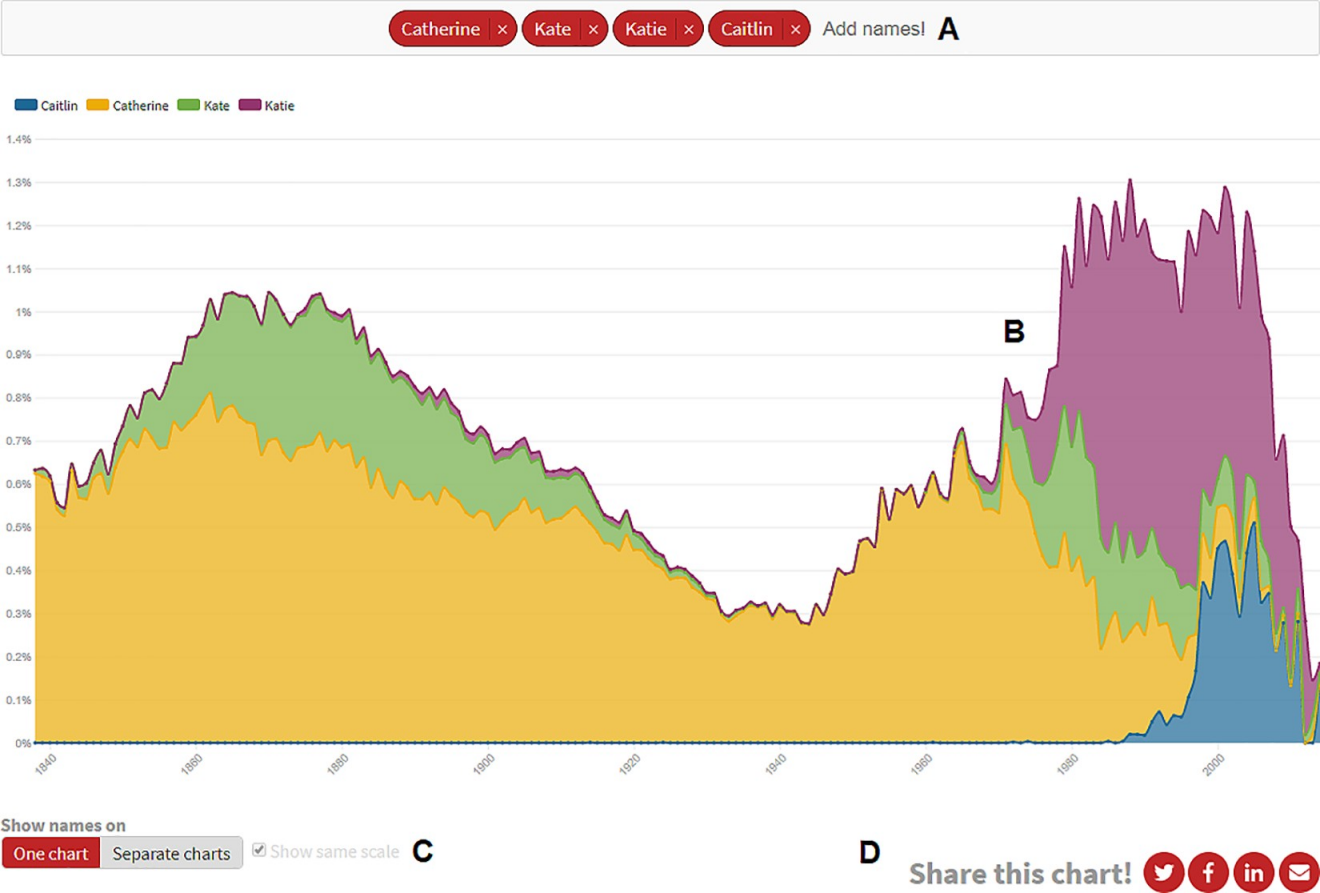
Web interface for visualising forename usage data. Forename usage profiles in the BMD dataset (S8 Table) are available to search online at http://demos.flourish.studio/namehistory. Names can be entered into a search box (A) to create line graphs (B). These graphs automatically update based on the number of names added to the search box, with data shown either on one chart or as separate charts, which in the latter case can be constrained to the same scale (C). Charts may be shared via numerous online platforms (D). Interface designed using Flourish data visualisation tools (http://flourish.studio).

The BMD forename usage profiles (S8 Table) are also available to search via an easy-to-use web interface at http://demos.flourish.studio/namehistory. This interface uses Flourish data visualisation tools (http://flourish.studio) to produce line graphs both for individual names or groups of names, and features dynamic graph rescaling, search autocompletion and the options to combine, split and share graphs. An example is illustrated in Fig 1.

All BMD name usage profiles are also available in S1 Dataset (hosted on the University of Edinburgh DataShare portal at http://dx.doi.org/10.7488/ds/2294), an archive containing paired ‘.csv’ files (an input format readable by Graphia Professional) and ‘.layout’ files, a text file format output by Graphia Professional that describes the characteristics of the network so that it may be replicated (described in fuller detail on the Graphia Professional support wiki at http://www.kajeka.com/wiki).

All data constitutes the outcome of text-mining analysis of the local BMD records–in accordance with the data usage statement, no original birth records are included in this publication or its associated supplementary material, and nor is it possible for records to be reconstructed from the data presented therein.

## Results

### Network analysis reveals successive vogues and fads in name use

**Fig 2:**
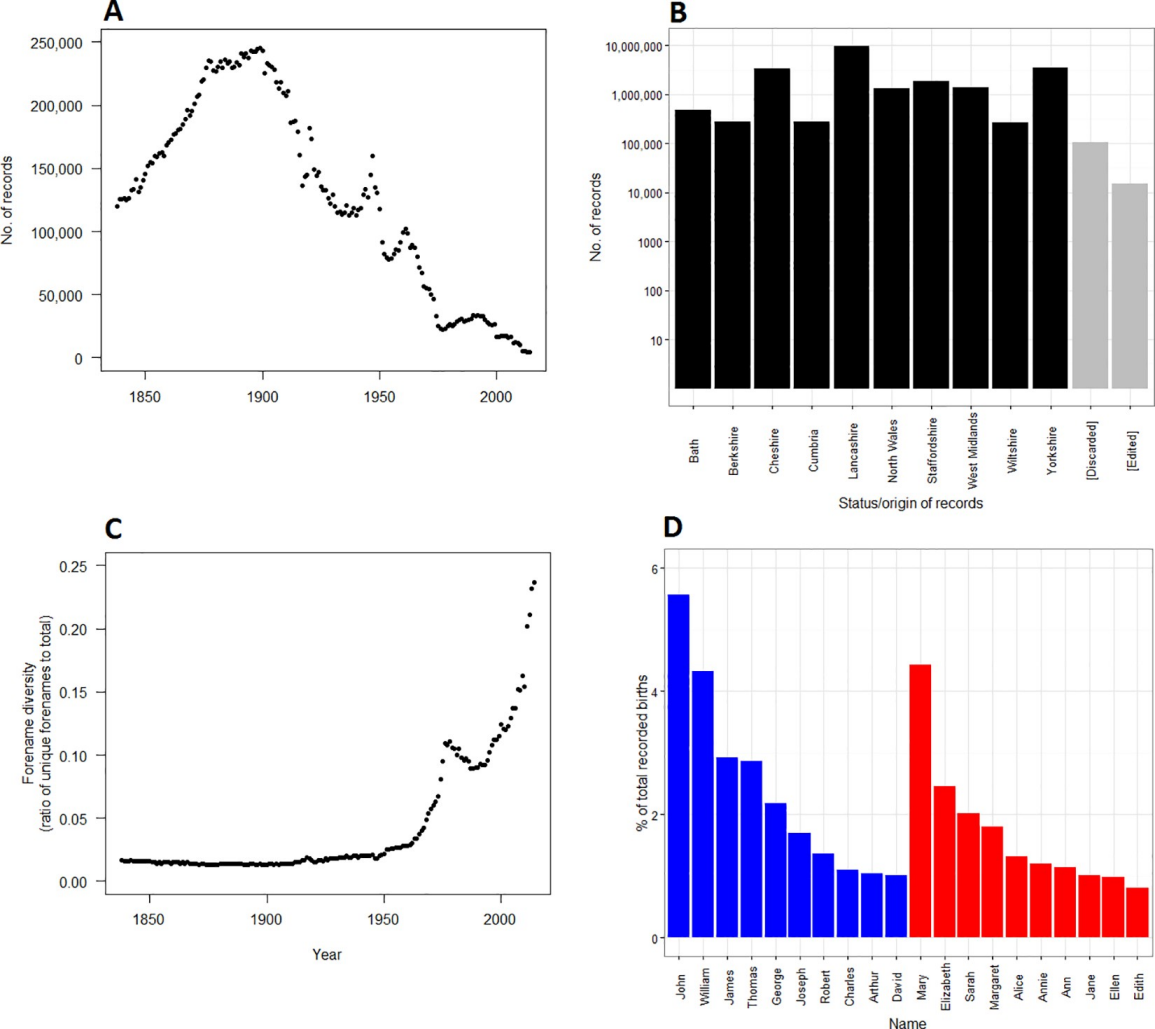
Overview of the primary data. **(A)** Number of usable records per year, and (B) per geographical region, along with the number of records edited and discarded. Edited records are those with typographic errors, and discarded records are those unrecognisable as names (detailed in S1 and S2 Tables, respectively). **(C)** Forename diversity per year, the number of unique forenames as a proportion of the number of births. **(D)** The top ten most popular male and female names across the entire dataset, ranked left to right. These rankings are affected by the disproportionately greater number of historical records. The most popular name in a given year is detailed in S6 Table.

Correlation graphs condense complex onomastic data into an accessible, and visually intuitive, format so that vogues in name use may be explored. To this end, we collected and curated an onomastic dataset of forenames and middle names drawn from the regional birth registers of England and Wales. An overview of the primary data, in terms of the number of records obtained per region and per year, and their associated diversity, is shown in Fig 2. Various features of this corpus, identifiable without network analysis, are discussed in S1 Text.

**Fig 3:**
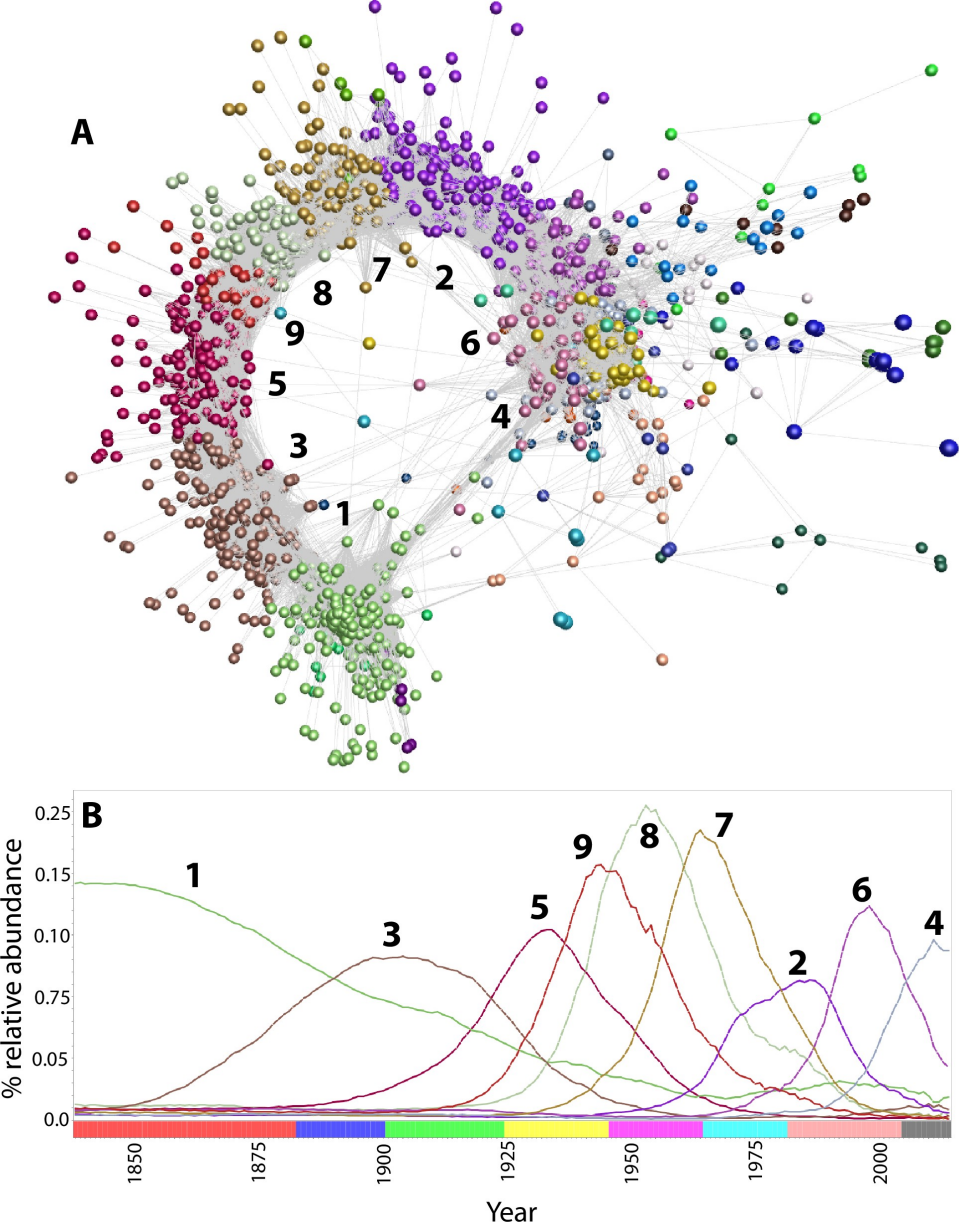
Forename usage in the English and Welsh birth registers (1838–2014). Forename usage represented as **(A)** a network graph, and **(B)** as relative abundance over time for nine clusters of names, the contents of which have similar usage profiles. For the network graph, a minimum Pearson correlation coefficient was applied of 0.6, i.e. those edges (correlations) that connect nodes (names) with a value lower than this are excluded. Overall, the graph contains 2835 nodes and 187,480 edges. Each set of coloured nodes represents a cluster of names with similar usage profiles. Selected clusters are numbered both in the network graph and the abundance plot. The contents of each cluster, and those names that do not form a cluster, are available as S12 Table. Name usage data is available as S8 Table. For ease of interpretation, the bar at the base of the abundance plot demarcates generations in arbitrary colours. Using colloquial generation names, from left to right: Georgian/Victorian (year of birth < = 1882), The Lost Generation (1883–1900), The Greatest Generation (1901–1924), The Silent Generation (1925–1945), The Baby Boomers (1946–1964), Generation X (1965–1981), Generation Y/Millenials (1982–2004), Generation Z (> = 2005).

The corpus of forenames is plotted as a network graph in Fig 3. This graph is a compact, information-rich representation of approximately two centuries of parental choice when it comes to naming their children, its elongated topology representing the continuum of naming vogues over time. Clusters comprise names that form local areas of high connectivity within the graph due to similar (correlated) usage profiles. Clusters are shown as groups of identically coloured nodes (the names within each cluster are detailed in S12 Table) and represent groups of names that rise and fall in popularity over the generations in a similar manner. Although this ‘wave’ pattern is, broadly speaking, consistent throughout history, contemporary vogues appear to be shorter lived.

A succession of clusters can be followed from left to right around the central structure of network graph, tracking the period of time from past (left) to present (right). In the bottom left of the figure, cluster 1 represents the cultural milieu of the UK in the 19^th^ century, containing (among others) the Old Testament male names Cephas, Enoch, and Theophilus, and female names Hephzibah, Tryphena, and Zilpah, alongside Christmas and Easter, Charity, Faithful, Mercy, Prudence, and Virtue. This is particularly notable as few names (c. 6%) in the Judeo-Christian scriptures–for which, presumably, many people were named–are female [25].

Other clusters reflect demographic changes in the UK throughout the 20th century. For instance, cluster 8 (peak usage c. 1953) contains a subset of names of Polish origin, including Andrzej, Bohdan, Danuta, Halina, Henryk, Jerzy, Ryszard, Stanislaw, and Zbigniew, and cluster 7 (peak usage c. 1963) names of Italian (Domenico, Giovanni, Guiseppe, Luigi, Salvatore) and Indian (Baljit, Jasbir, Karan, Manjit, Parmjit, Surinder) origin (S12 Table). These clusters are consistent with historic waves of immigration to the UK after the Second World War, traditionally marked by the Polish Resettlement Act 1947 and Indian Independence Act 1947. Cluster 5 (peak usage c. 1933) contains subsets of names of Irish origin (such as Aileen, Aline, Alma, Eileen, and Sheelah) and Welsh origin (such as Buddug, Cledwyn, Gwynedd, Morfydd, and Olwyn), consistent with emigration from the Irish Free State and associated civil war (1922–1923), and the growth of Welsh nationalism throughout the 20th century, respectively.

Towards the right of Fig 3 (the 21st century), there are a greater number of smaller clusters of nodes, representing the greatly increased diversity of contemporary name choices. For instance, clusters 10 (55 names), 11 (49 names), 12 (46 names), 13 (46 names) and 14 (20 names) are typified by contemporary choices–such as Pippa (cluster 10), Kyla (cluster 11), Troy (cluster 12), Aurora (cluster 13) and Astrid (cluster 14)–alongside names reflecting ongoing demographic changes to the UK population, such as those of Islamic origin: Nabeel (cluster 10), Iqra (cluster 11), Nafisa (cluster 12), and Khadija (cluster 13). Whilst names frequently provide a clue as to a child’s ethnicity, definitively assigning an individual to a specific country of origin based on name alone is a non-trivial problem of interest in the study of social integration and mobility [26,27]. Despite a common ethnocultural origin, these names are also distributed among several clusters. This is because the increased diversity of contemporary name choices results in fewer births, in absolute terms, registered with each name. Consequently, in any given year, the use of each name is more subject to chance.

**Fig 4:**
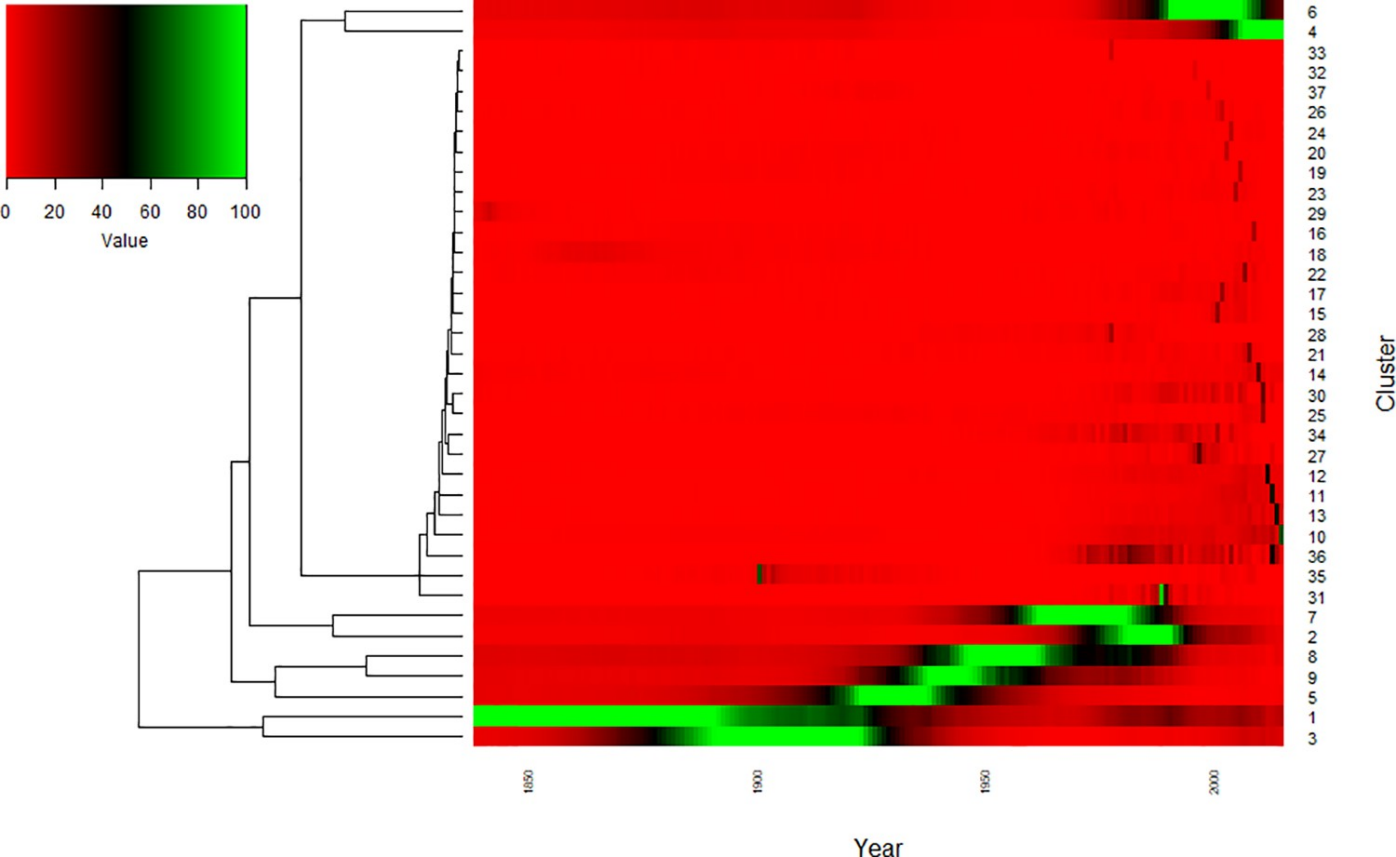
Clusters of forenames with correlated usage profiles in the English and Welsh birth registers (1838–2014). Average abundance (% usage) of all forenames per cluster, represented as a heatmap. Values are scaled according to the maximum abundance of each cluster in a given year. The contents of each cluster are available as S12 Table. Name usage data is available as S8 Table. Clusters are numbered arbitrarily, in descending order of size.

Trends in the BMD dataset can also be visualised as a heatmap (Fig 4). While this provides an alternative representation of the ‘wave’ pattern of name usage (Fig 3), it also highlights the peak usage of smaller clusters. For instance, cluster 29 contains 6 names, each of which is a spelling variant more commonly used c. 1850 but rare in contemporary records: Cathrine, Ellinn, Feargus, Hesther, Jenney, and Margarett.

Fads, short-lived increases in a name’s popularity driven by events of the time, can be associated with, for instance, a specific popular public figure, real or fictitious. Fads are usually isolated events and so rarely form clusters. A notable exception is cluster 35 (see Fig 4), which contains 4 names (Baden, Hector, Redvers, and Pretoria) all of which peak in popularity in 1900. These names can be associated with the Second Boer War (1899–1902). Redvers is likely a reference to the initial commander of the British forces, General Redvers Buller, and Baden is likely a reference to Colonel Robert Baden-Powell, the British commander of the besieged town of Mafeking (now called Mahikeng), an event which attracted considerable publicity due to the presence in the town of the then Prime Minister’s son. The association of Hector with this spike in popularity is less easy to assign with any certainty but could be a reference to Colonel (later Major-General Sir) Hector ‘Fighting Mac’ MacDonald, a popular figure who became famous after the 1898 Battle of Ombdurman. The city of Pretoria was captured by the British in 1900. Naming fads are discussed in further detail in S1 Text, with contemporary UK naming fads seemingly inspired more strongly by popular culture.

### The use of name derivatives

The grouping of names in a cluster invites speculation: are there shared characteristics that could explain their shared popularity (that is, their correlated usage profiles)?

**Fig 5:**
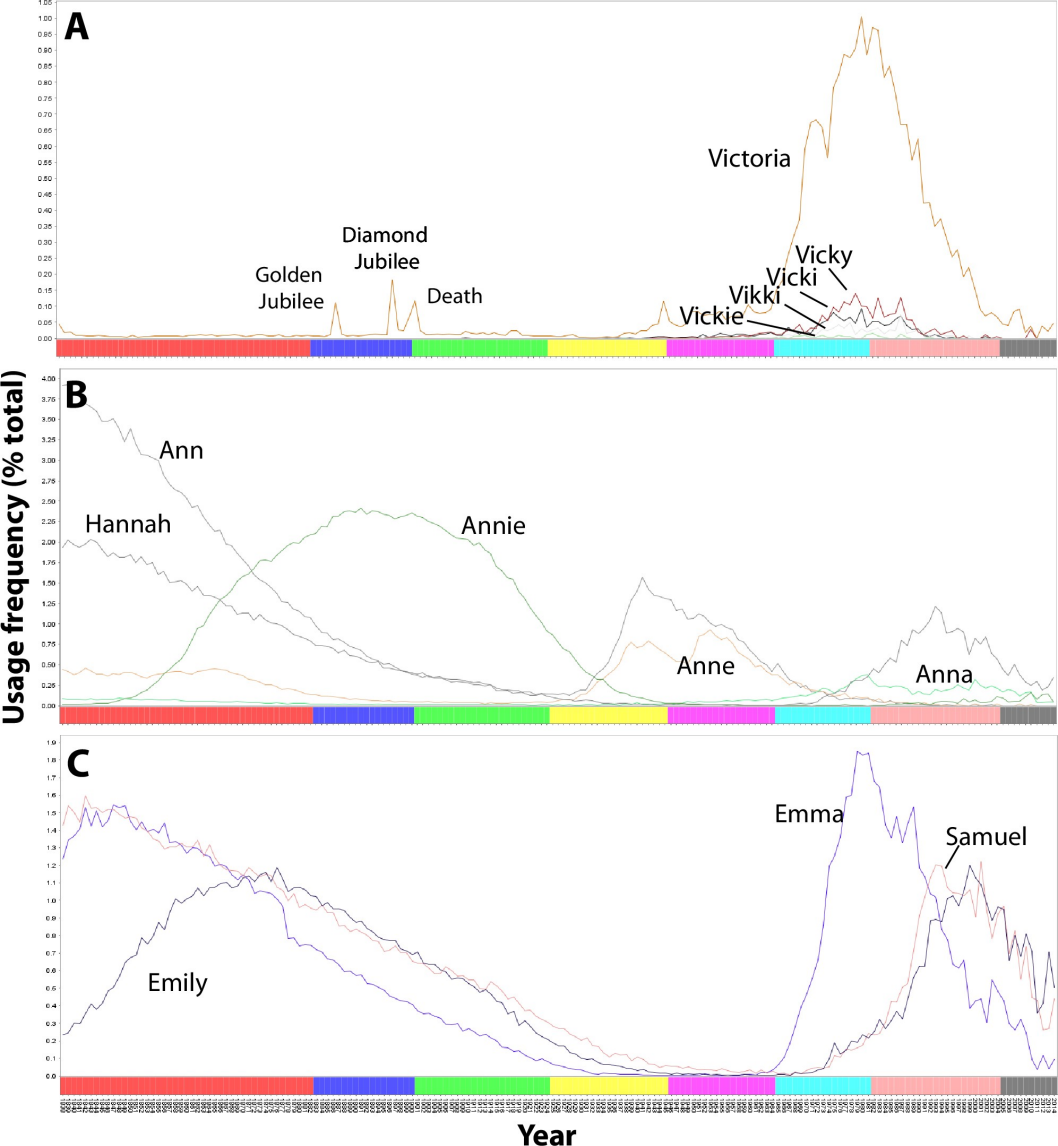
Usage profiles of individual names, illustrating various naming trends. **(A)** Vicki, Vickie, Vicky, Victoria, and Vikki (name variants can have correlated usage profiles), **(B)** Ann, Anna, Anne, Annie, and Hannah (the popularity of name variants can fluctuate in inverse proportion to their prevalence), and **(C)** Emily, Emma, and Samuel (historically popular names can reoccur as contemporary choices). In panel A, the golden jubilee, diamond jubilee and death of Queen Victoria are indicated (1887, 1897 and 1901, respectively), each event briefly coinciding with a rise in the use of the name.

For instance, spelling variants and name derivatives often cluster together. Cluster 1 (peak usage c. 1843) contains Rosanna, Rosannah, Roseannah, and Rosehannah, cluster 6 (peak usage c. 1998) Abbey, Abbi, Abbie, Abby, and Abigail, and cluster 2 (peak usage c. 1983) Vicki, Vickie, Vicky, Victoria, and Vikki (Fig 5A). Other name derivatives, however, do not cluster, instead showing different patterns of usage, rising and falling in popularity in a manner opposed to each other: one name waxing in popularity as the other wanes. This is particularly apparent for variants of especially popular names, such as Rose (cluster 3), Rosemary (cluster 9) and Rosie (cluster 4), and for Ann (cluster 1), Anna (cluster 2), Anne (cluster 8) and Annie (cluster 3), as illustrated in Fig 5B. Although one of the most popular names in the 19^th^ century, Ann declined in use towards the 20^th^ century as that of Annie increased. As Annie declined in popularity (to negligible use by the 1950s), the prevalence of Ann increased–alongside Anne, a previously uncommon variant. Neither variant remained widely used, however–towards the end of the 20^th^ century, the most popular variants became Hannah (which was also previously popular in the 19^th^ century) and Anna. Other usage profiles are bimodal, suggesting the recurrence of certain names over time regardless. Most notably, Emily, Emma and Samuel were each registered in c. 1% of births in the Victorian era, but fell to negligible use by the 1950s, only regaining popularity towards the present day (Fig 5C).

### Contemporary name use data

**Fig 6:**
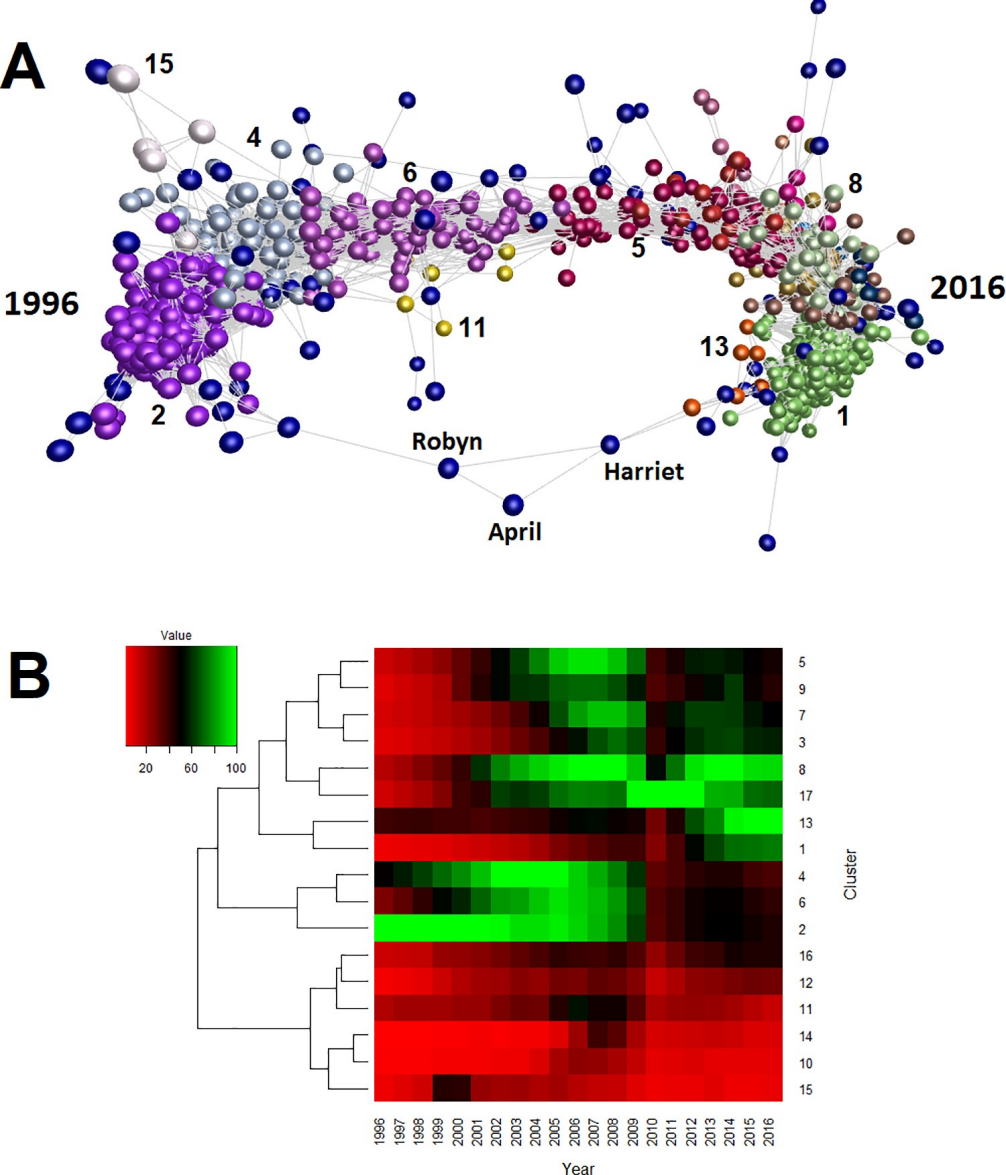
3D network graph and heatmap representations of 1846 forenames from the UK Office for National Statistics birth records (1996–2016). **(A)** The network graph contains 1846 nodes (names) and 221,251 edges (nodes with Pearson’s *r* ≥ 0.85). Three labelled names–April, Harriet, and Robyn–have clear bimodal usage profiles, peaking in popularity both at the beginning (1996) and end (2016) of the dataset. Nodes coloured dark blue (including April, Harriet and Robyn) are those not assigned to a cluster. **(B)** Average abundance (% usage) of all forenames per cluster, represented as a heatmap. Values are scaled according to the maximum abundance of each cluster in a given year. Selected clusters are numbered both in the network graph and the heatmap. The contents of each cluster, and those names that do not form a cluster, are available as S14 Table. Name usage data is available as S13 Table.

To extend the above analyses using contemporary data, we obtained records of all live births in the last twenty years from the UK Office for National Statistics (see Materials and Methods). We calculated the usage of each name as a proportion of the total births per year (S13 Table), constructed a network graph (Fig 6A) and heatmap (Fig 6B) after excluding names with fewer than 500 registered births overall, performed a cluster analysis on the network graph (S14 Table), and recorded both the number of uniquely identified names and associated forename diversity (S15 Table). The graph of the ONS dataset has a similar topology to that of the local BMD dataset (Fig 3), and also contains names a small subset of names with bimodal usage profiles (April, Harriet, and Robyn)–these names recur in popularity at two distant points in time, connecting the two ends of the graph (individual usage profiles for these names are available at http://names.darkgreener.com/-april, http://names.darkgreener.com/-harriet, and http://names.darkgreener.com/-robyn, respectively). These bimodal profiles are similar to that of (for example) the contemporary name choices Emma, Emily and Samuel, which were each popular in the Victorian era (Fig 4C). In this respect, the ONS dataset can be considered a higher resolution subset of the local BMD data, but covering a far shorter period. While clusters 1 and 2 of the ONS dataset represent names that, respectively, show an average increase and decrease in usage over the 20 year period, many of the other clusters distinguish rises and falls in popularity about a specific year: 2002 (cluster 4), 2003 (cluster 6), 2006 (cluster 5), 2009 (cluster 7), and 2010 (cluster 3) (S14 Table). In general, this suggests that naming trends on a smaller scale (the ONS dataset) mirror those on the large (the BMD dataset), with both datasets showing ‘wave’ patterns of relative abundance and having an elongated topology to their network graph. As with the BMD dataset, clusters derived from the ONS dataset can show fads as well as vogues. For instance, cluster 17 (see Fig 6B), which contains 4 names, is dominated by the sudden popularity in 2010 of the name Maisie, along with two variants, Maisy and Maizie (the fourth name in this cluster, Kaiya, is likely a spurious correlation as it has a low overall frequency).

The most notable large-scale trend in both the BMD and ONS datasets is that of increased forename diversity (discussed further in S1 Text). While it is tempting to attribute this increase primarily to changes in the ethnic diversity of the UK population, this is not a wholly satisfying explanation for the trend. This is because demographic change should increase entire subsets of culturally associated names, such as the Polish, Italian and Indian names seen in clusters 7 and 8 of the BMD dataset (discussed above). Towards the present day, however, such clearly defined subsets of names are not readily apparent.

The ONS dataset has far higher resolution than the BMD dataset for recent name usage, being a complete catalogue of all UK births since 1996. This dataset also shows a year on year decrease in the proportion of records registered with the most popular name, a year on year increase in forename diversity (the ratio of the number of unique forenames to the total number of births per year), as well as an increase in the proportion of names uniquely registered in only one year (S1 Fig and S15 Table). Notably, approximately 65% of the names in the ONS dataset are registered to fewer than 10 individuals in a given year, and approximately 4% of names are recorded in only 1 of the 20 years (S15 Table). These names do not often cluster, being dissimilar in usage profile to most other names.

Many of these unique names are novel coinages–that is, derivatives of existing names–rather than an outside introduction to the pool of possible choices (discussed further in S1 Text). For instance, there has been an increase in the proportion of hyphenated forenames (that is, combinations of two existing names) from 2.5% of the total number of unique names registered in 1996 to 9.1% in 2016 (the usage profiles of all hyphenated forenames, showing a clear upward trend, are available at http://names.darkgreener.com/-.*-.*), as well as an increase in the number of names with variant endings (such as Hollee, Holley, Holli, Hollie and Holly) (S1 Fig).

This suggests that alongside demographic change in the latter half of the 20th century, which broadened the pool of possible names, there has also been a societal shift towards name distinctiveness: when choosing a name in the 21st century, relative rarity appears highly desired.

## Discussion

A child’s name may be chosen by reference to the parent’s values, expectations and desires. Why, then, do certain names experience vogues–to cycle in and out of fashion between generations? It can safely be assumed that the societal expectations of one age, and their associated values, will differ from another–and yet certain names recur over time regardless, likely because their connotations change too. It is tempting to speculate that cultural changes underlie these observations. For example, contemporary use of the Old Testament names Jemima and Kezia–two of the three daughters of Job–is less likely to be an explicit reference to their biblical counterpart. Although decreasingly popular throughout the 20^th^ century, both names were used in the 19^th^ century (being a less secular time, this is ostensibly in reference to their namesakes), as was–to a lesser extent–the third daughter’s name, Keren-happuch (all three names are found in cluster 1 of the BMD dataset; S12 Table). In the BMD dataset, Keren-happuch has been unused as a name since 1894 (it is absent from the ONS dataset). It is reasonable to suppose biblical names lose prominence and appeal in more secular times, with subsequent disconnection from these original associations. By the latter half of the 20^th^ century, the name Jemima was perhaps more widely associated in the UK not with the Old Testament but with Jemima Puddleduck, a character imagined by the children’s author Beatrix Potter. An alternative possibility is that the 19^th^ century popularity of, for example, Kezia, was in essence aesthetic rather than honorific, perhaps similar to the contemporary use of Esther, Ruth and Mary–the biblical namesake may in each case be irrelevant (or unknown) to the parent choosing this name. There can be little question that the cultural influence of Christianity has altered in the UK over the last century, and that there has been declining use in many names derived from the Bible (for instance, in the ONS dataset, Kezia is not even in the top 500 most popular female names, being registered only 890 times in 20 years).

The network analysis methods applied here, to highlight vogues and fads in name use over time, can be used on any large numeric dataset, and provide both a means of visualising big data and of analysing it in a hypothesis-free manner. In doing so, we open new avenues of exploration.

For instance, we may consider the question: what–in general–predicts naming vogues? Bimodal name usage profiles–such as those for Emma, Emily and Samuel, which peak in popularity in the late 19th and late 20th centuries–could be explained using the preference-feedback hypothesis of Colman, *et al*.: that the naturally occurring frequency of exposure to a stimulus, i.e. a name, determines the degree to which it is favoured [28]. This predicts that name choice is to some extent a function of exposure–popular names are liked because they are popular, and become more popular because they are liked (a previous study has shown that names are also more likely to become popular if phonetically similar names have been recently popular too [13]). This positive reinforcement holds only up to a certain point, however–beyond this, names decline in popularity because they may be perceived as over-used. A social pressure may then apply to avoid certain–previously popular–names, perhaps as a desire to distinguish the child from other individuals in the parents’ social network (who are themselves more likely to have popular names), to otherwise prevent the child from being considered ‘common’ or ‘ordinary’, or to affirm the child’s membership of the present generational cohort (within which common ‘old person’ names–those associated with the grandparental generation–may be avoided as forenames). Name choices may otherwise be subject to random drift, with changes in their frequency explained by a simple model–because individuals randomly copy names from each other, repeated sampling of a population over time drives some names to higher frequencies and causes some names to be lost (such as the comparatively low-frequency Keren-happuch, discussed above) [29,30]. The preference-feedback model is superficially similar to the drift model in that the popularity of a name is related to its frequency in the population. However, the drift model assumes all names are value-neutral, whereas the preference-feedback model suggests that certain names have (or can acquire) greater intrinsic value than others: the value of relative rarity.

Rarer names, by virtue of being rare, can allude to the originality or distinctive nature of the bearer (i.e. how a parent views a child). This has various social benefits (reviewed in [31]): uncommon names have been positively associated with academic performance [32], professional standing [33] and assessments of artistic creativity [34]. In general, rarer names emphasise a child’s individuality, and shape a desirable image of their abilities or works [34].

The preference-feedback hypothesis suggests that rarer names are chosen primarily because they are rare–but that over time this increases their exposure, decreasing their appeal. This is consistent with the oscillating usage profiles illustrated in Figs 1, 3 and 5, the observation that contemporary name choices alter more rapidly in popularity than in previous generations, and with the proliferation of rare name variants towards the present day. For instance, approximately 4% of names are registered in only one year of the two-decade ONS dataset, such as the rare variants Abbiegayle (only recorded in 1998), Abagael (1999), Abygayle (2000), Abaigael (2004), and Abbygael (2013). This suggests that in the present day UK, one of the more desirable properties of a name is its distinctiveness. This coincides with upward trends in uncommon name choice observed in contemporary China [35], Japan [36] and the United States [37], and related to increased individualism and the ‘need for uniqueness’: “a positive striving for abnormality relative to other people” [38]. In this respect, spelling variants can add an acceptable degree of ‘abnormality’ to certain names. For instance, the comparatively rarer variants Rebekah, Aimee and Ashleigh (cluster 6 of the BMD dataset, peak usage c. 1998; S12 Table) each rise in popularity after the more commonplace Rebecca, Amy and Ashley (cluster 2 of the BMD dataset, peak usage c. 1983).

As illustrated by the example of the three daughters of Job (see above), name choices in the UK appear simultaneously influenced both by external social forces (such as the varying cultural dominance of Christianity over time) and internal mechanisms (such as via the drift and preference models of cultural change which, respectively, predict why the unknown Keren-happuch is not in contemporary use and why the uncommon Kezia is).

It is not possible to definitively predict the motivations for a given name choice and reasonable to believe no single model of cultural evolution will satisfactorily explain the volatile dynamics of all name usage profiles. Indeed, cultural evolution differs from genetic evolution in one critical sense: the act of transmission of a cultural trait (such as a name) can itself affect the mechanism of transmission (if transmission is popularity-dependent) [17].

Historically distinctive changes in UK naming occurred with the social and economic upheaval of the Industrial Revolution, with little stability seen in naming patterns since [39]. The speed with which contemporary name choices fall in and out of favour likely reflects their more extensive exposure. In the present day, with its comparatively greater range of media and freedom of travel, social networks are not only larger but more globally (and virtually) distributed. In this respect, we can predict increasingly short periods of time before a contemporary name is considered ‘over-used’ and so starts to fall out of vogue. While the freer movement of people throughout the 20th century has, by the present day, expanded the pool of possible names, so too has the social freedom to coin novel variants of existing names. In today’s world of ubiquitous media exposure, beliefs about popularity (that is, beliefs made in an environment of relative social freedom) may be self-fulfilling: ‘fad names’ are short-lived because people believe they will be short-lived, reducing their subsequent appeal [15].

In summary, here we apply tools and techniques originally devised for the biosciences to onomastics. In particular, we demonstrate the use of network graphs for condensing large-scale name datasets thereby allowing the analysis of long-term cultural, social and demographic changes within the UK. This approach is sufficiently high-resolution as to resolve short-lived contemporary naming trends.

## Supporting information

S1 Text General features of the BMD and ONS corpora of names. (DOCX)

S1 Table Typographical changes made to the BMD corpus of names. (XLSX)

S2 Table Records excluded from the BMD corpus as they are unrecognisable as a complete name. (XLSX)

S3 Table Rank order of forenames in the BMD corpus, by number of registered births per year. (XLSX)

S4 Table Rank order of middle names in the BMD corpus, by number of registered births per year. (XLSX)

S5 Table Rank order of names in the BMD corpus, by total number of registered births (across 177 years). (XLSX)

S6 Table Summary of the BMD corpus: Number of usable birth records per year, forename diversity, proportion of records with a middle name and the most popular fore/middle name per year. (XLSX)

S7 Table Co-occurrence of forenames and middle names in the BMD corpus. (XLSX)

S8 Table Usage of forenames in the BMD dataset, as a percentage of total registered forenames per year. (XLSX)

S9 Table Usage of middle names in the BMD dataset, as a percentage of total registered middle names per year. (XLSX)

S10 Table Usage of forenames in the BMD dataset, as the absolute number of registered forenames per year. (XLSX)

S11 Table Usage of middle names in the BMD dataset, as the absolute number of registered middle names per year. (XLSX)

S12 Table Clusters of names with similar usage profiles in the BMD dataset, obtained after network analysis of forename usage (as a percentage of total registered forenames per year). (XLSX)

S13 Table Usage of forenames in the Office for National Statistics dataset, as a percentage of total registered forenames per year. (XLSX)

S14 Table Clusters of names with similar usage profiles in the Office for National Statistics dataset, obtained after network analysis of forename usage (as a percentage of total registered forenames per year). (XLSX)

S15 Table Number of unique forenames, and forename diversity, in the Office for National Statistics dataset. (XLSX)

S1 Fig Overview of the ONS dataset. Overview of the ONS dataset, showing (A) the forename diversity per year (the number of unique forenames as a proportion of the number of births), (B) the percentage of names only registered in this year, (C) the percentage of records registered with the most popular name of that year, (D) the percentage of records registered with any of the 10 most popular names of that year, and (E) the percentage of records registered with a hyphenated forename (counting only the 13 most common second names in a hyphenated name). Graph (F) uses a subset of 61 ‘root’ forenames in which all 5 common endings (-ee, -ey, -i, -ie, or -y) have been registered at least once in the dataset. The figure shows the percentage of the total possible name combinations that are registered this year, i.e. the number of names used out of 61x5 = 305 possibilities.
(TIF)
