# Supplementary material for: Network analysis of the social and demographic influences on name choice within the UK (1838-2016)
Source: PLoS One. 2018 Oct 31;13(10):e0205759. doi: 10.1371/journal.pone.0205759 (PMC6209202; doi:10.1371/journal.pone.0205759)
Supplement: S1 Text — (DOCX) [file pone.0205759.s001.docx]

**Supplementary Text**

This supplementary text discusses general features of the name datasets used for this study: the local birth, marriage and death (BMD) records from England and Wales, spanning the years 1838 to 2014, and an Office for National Statistics (ONS) dataset spanning the years 1996 to 2016.

***Expanding middle name usage***

The local BMD corpus spans the majority of time for which middle names have been commonly used in the UK, and so captures a variety of trends.

The custom of giving a child more than one name began in Renaissance Italy [[1](#_ENREF_1)] but was slow to become established in England: by 1800, only approximately 10% of the population had more than one name [[2](#_ENREF_2)]. This is reflected in the proportion of birth records with middle names, rising from approximately 20% in 1850, to 40% in 1900, 60% in 1950 and 90% by 2000 (S6 Table). For birth records with middle names, there also appears an upward trend over time in the number of middle names registered per individual – the average number of middle names per birth record, for birth records with one or more middle name, also increases throughout the 20^th^ century (S6 Table), with a greater proportion of contemporary births having multiple (typically, 2 or 3) middle names.

The majority of contemporary records contain only one middle name, with a few records having many. Many of these extreme cases are related to football. There are several newspaper reports of people named for the surnames of every player in the football team supported by a parent (sometimes including the surnames of managers and substitute players, as well as all 11 members of the team). Teams so honoured include, in 1965, Liverpool F.C. (in a record with 15 middle names: *St John Lawrence Lawler Byrne Strong Yeats Stevenson Callaghan Hunt Milne Smith Thompson Shankly Bennett Paisley* [reported in *The Liverpool Echo*; https://tinyurl.com/y923x33f]); in 1967, Rangers F.C. (11 middle names: *Martin Johansen Provan Jardine McKinnon Greig Henderson Willoughby Miller Smith Wilson* [reported in *The Daily Record*; https://tinyurl.com/ydgq32y6]); in 1992, Leeds United F.C. (12 middle names: *Andrew Lukic Newsome Fairclough Whyte Dorigo McAllister Batty Strachan Speed Chapman Cantona* [reported in *The Daily Mail*; https://tinyurl.com/ppnnecv]); and in 2011, Burnley F.C. (the first name, Jensen, is given for the team goalkeeper, with 13 middle names honouring the other players: *Jay Alexander Bikey Carlisle Duff Elliot Fox Iwelumo Marney Mears Paterson Thompson Wallace* [reported in *The Daily Mail*; https://tinyurl.com/5rjb5jm]).

As the BMD dataset is a not a full population sample, it does not contain these specific birth records. However, it does contain a 1969 birth record with 11 middle names (*Aston Best Kidd Charlton Law Stiles Stepney Sadler Burns Morgan Crear*), which might otherwise pass without notice – these are all members of the Manchester United football team (*Crear* is likely a typographical error in the transcription of the record, as this should be the name of midfielder Pat Crerand). By contrast, historic records in the BMD dataset have, at most, 7 or 8 middle names – but unlike contemporary records with large numbers of middle names, the middle names appear to be forenames, not surnames. The most extreme cases include, in 1850, *Albert Anson Granvilly Savaran Lorenzo Thambor Oliver Kinnersley Watson*, in 1869, *Hannah Maria Matilda Sophia Julianna Georgina Lucretia Cinderella Martave Cotgreave*, in 1875, *Percival Tristram Leofric Heald Leopold Augustus Ivan De Leland Leyland*, and in 1885, *Beatrice Margaret Georgina Selina Ruth Alma Mabel Adelaide Augusta Collier*. These records are particularly notable as historically, it was common for a surname, often a mother’s maiden name, to have been used as a middle name.

***Forenames as the transferred use of surnames***

We can speculate that the increasingly widespread adoption of middle names, many of which were originally surnames, facilitated the eventual transfer of some names to a first name position. To identify which forenames are more likely to be transferred uses of surnames, we calculated the ratio of the number of times the name is used in either position (S5 Table). Larger values are suggestive of transferred surname usage, but this must be interpreted with caution – the ratio can be skewed by a low absolute number of forename records. For example, *Riley* (from the Irish Gaelic ‘valiant’) is used 45 times more as a surname than a forename: 39,856 to 873 records, respectively (it is also registered 935 times as a middle name). Given this high ratio, it is reasonable to believe modern uses of this forename reflect a transferred surname usage over time, given the large number of forename records. By contrast, the surname *Jones* – recorded 435,219 times – is registered 5432 times as a middle name, but only 76 times as a forename, a comparatively low number.

In general, many of the names with the highest surname-to-forename ratios also have high middle name-to-forename ratios, for instance *Cooke*, *Cox*, *Hunt*, *Poole* and *Parr*, each of which are recorded once as a forename, hundreds of times as a middle name and thousands of times as a surname. This pattern is consistent with the adoption of pre-marital surnames as middle names upon marriage [[3](#_ENREF_3)], and their propagation as family names thereafter.

For the set of middle names registered per year, we calculated the number of times each is recorded as a surname and the number of times each is recorded as a forename, expressing this as a ratio (S6 Table). Prior to 1850, registered middle names were on average 25 times more likely to also be registered as a surname, rather than a forename. However, by 1900, this ratio falls – and remains – far below 1, suggesting that towards the present day the preference for choosing middle names is to choose them from a pool of forenames, rather than to choose a (familial) surname. This is likely related to a contemporary preference for adopting double-barrelled surnames upon marriage, which combine a pre-marital surname with the surname of the spouse. Children of this union may then have one parent’s pre-marital name as part of their surname, rather than – historically – as a middle name, if at all.

***Co-occurring forenames and middle names***

Considering only birth records with one forename and one or more middle names, we calculated the ratio of the number of times each forename is registered with a given middle name to the number of times that forename was registered with any middle name (S7 Table). This distribution of ratios has a long tail. Of the 6059 forename/middle name pairs considered, the majority (43%, i.e. 2591 pairs) have ratios < 1, suggesting that the forename does not co-occur with any particular middle name all that frequently. By contrast, some middle names disproportionately co-occur with certain forenames. With the exceptions of *Albert Edward* (35%) and *William Henry* (23%), the top 50 most highly co-occurring forenames and middle names are female. At the most extreme end of the distribution, the forename *Gemma* co-occurs with the middle name *Louise* in 47% of cases (for the spelling variant *Jemma*, 41%), with similarly high co-occurrences of *Amanda Jane* (40%), *Claire* *Louise* (39%), *Edna May* (33%), *Donna Marie* (32%) and *Mair Eluned* (31%). These ratios are not adjusted for the age of the record. Consequently, for many forenames, their co-occurrence with middle names will likely be skewed by the historic popularity of more traditional names (noting that the diversity of names was also, historically, lower). This is especially notable for each of the forenames *Betsy*, *Betsey*, *Beverly*, *Carol*, *Carole*, *Eliza*, *Elizabeth*, *Esther*, *Martha*, and *Sarah*, all of which co-occur with *Ann* in > 30% of cases (prior to 1850, *Ann* accounted for approximately 4% of all registered forenames).

***Atypical middle names***

In the BMD corpus (S5 Table), numerous names – that are not also surnames (see above) – are found only in the middle position. However, this is uncommon: the majority of middle names are also used as forenames (particularly in contemporary records) or, if not, are often transferred surnames (particularly in historic records). Nevertheless, as the middle name is less frequently spoken, written or required, it can be used in creative fashion. Atypical middle names have been used to reference historic figures (*Aristotle*, *Charlemagne, Descartes, Lenin, Marx, Pasteur, Plato, Robespierre, Socrates, Stalin, Trotsky, Voltaire*), historic occasions (*Armistice* and *Versailles* [in records from 1918-1923, the end of the First World War], *Coronation* [in 9 middle name and 4 forename records from 1902, the year of the coronation of King Edward VII], *Jubilee* [in 77 records from 1887, and 33 from 1897, the Golden and Diamond Jubilees of Queen Victoria, respectively]), regional locations (*Derwentwater*, *Snowdonia, Staffordshire, Windermere*), cities (*Delhi*, *Dublin*, *Lisbon*, *London*), countries (*Belgium*, *Germany*), battlefields and military campaigns (*Balaklava*, *Inkerman* and *Sebastopol* [in records from 1854, i.e. contemporaneous with the Crimean War], *Omdurman* [in records from 1899, i.e. contemporaneous with the Mahdist War], *Elandslaagte*, *Ladysmith* and *Mafeking* [in records from 1899-1902, i.e. contemporaneous with the Second Boer War], *Gallipoli* [in one record from 1917; one record each of *Dardanel* and *Dardanella* may also be found in 1915 and 1916, respectively], *Jutland* [in records from 1916], *Passchendaele* [in records from 1917 and 1918], *Somme* [in records from 1916 to 1919], *Ypres* [in records from 1914 to 1920]), mythological creatures and peoples (*Amazon*, *Centaur*, *Valkyrie*), deities (*Quetzalcoatl*), the Bible (*Leviticus*, *Maccabees*, *Proverbs*), fictional characters and popular culture (*Ivanhoe* [from the eponymous novel by Walter Scott], *Kal-el* [from the Superman comic series], *Tinkerbell* [from the novel ‘Peter Pan’ by J.M. Barrie], *Tintin* [from the eponymous Belgian comic series]), professional sportspeople (*Cantona* [after the Manchester United footballer; in records from 1993 to 2002]) and, presumably, familial occupations (*Scrivener*, *Shoesmith*, *Stonehewer*, *Ironmonger*, and *Makepeace* [a mediator]).

The parental perspective on the newborn may also be reflected in name choice (*Delight*, *Heavenly*, *Miracle*, *Purejoy*, *Treasure*), as could their aspirations for the child – names include aristocratic or royal titles (*Baron, Czarina*, *Emperor*, *Empress*, *Highness*, *Marquis*, *Monarch*, *Pharaoh*), military ranks (*Admiral*, *Captain*, *Colonel*, *General*, *Major*, *Officer*, *Sergeant*), occupations with high social status (*Bishop*, *Cardinal*, *Chaplain*, *Doctor*, *Evangelist*, *Judge*, *President*, *Primate* [i.e. Archbishop], *Seneschal*, *Vicar*), desirable accolades (*Champion*, *Patrician*, *Saint*) and attributes (*Bold*, *Constant*, *Faithful*, *Gallantry*, *Hopeful*, *Joyful*, *Obedience*, *Patient*, *Peaceful*, *Pious*, *Pleasant*, *Prudence*, *Sensitive*, *Silence*, *Temperance*, *Thankful*, *True*, *Virtue*, *Winsome*, *Wise*, *Wonder*, *Wonderful*).

In addition, both intangible properties (*Midnight*, *Stardust*, *Sunbeam*, *Sunrise*, *Sunshine*, *Twilight*) and natural ephemera (*Rainbow*, *Rosebud*, *Snowdrop*) could have been given as names for their aesthetically pleasing connotation. Middle names may also conceal social comment – for instance, one birth registered in the 1960s has a surname of Nigerian origin (*Emeruwa*) and the middle name *Englishgirl*. Although notable by their rarity, the motives behind these name choices are wholly speculative.

***Naming fads***

Many examples of naming fads (sudden and short-lived increases in popularity) are apparent in both the BMD and ONS datasets, which can often be associated with simple, singular, explanations of their cause. For instance, in 1987, the Australian actor and singer Kylie Minogue became famous in the UK via the soap opera *Neighbours*, resulting in relatively increased usage of her otherwise rare name. Of the 270 total records of *Kylie* in the BMD dataset, 158 (59%) are registered in the three year period of 1987-1989 (S10 Table). After network analysis of the BMD dataset, cluster 31 (S12 Table) can be seen dominated by an almost 15-fold increase in the popularity of *Kylie* in 1988. (Of the 3 other names in this cluster, one is a plausible variant, *Kaylie*, although the other two, *Les* and *Elon*, are likely spurious correlations as both have an overall low frequency).

Similarly sharp peaks in name use can be related to the debut albums of pop singers beginning their rise to fame, such as for *Britney* (after Britney Spears; name usage peaks in 1999 [http://names.darkgreener.com/#britney] at 314 registered births, with her debut album released in January of that year), *Rihanna* (known professionally by this mononym; usage peaks in 2007 [http://names.darkgreener.com/#rihanna] at 281 births, debut album 2005) and *Miley* (after Miley Cyrus; usage peaks in 2009 [http://names.darkgreener.com/#miley] at 522 births, debut album 2007). As with cluster 31 of the BMD dataset, which is dominated by the popularity of *Kylie*, cluster 15 of the ONS dataset (S14 Table) is dominated by the popularity of *Britney*.

A particularly notable example is of the Colombian singer *Shakira* (known professionally by this mononym), whose first English language album was released in November 2001, 10 years after her professional debut. This popularised her name – already known to a Latin American audience – to a UK audience, with a corresponding usage peak in 2002, at 258 registered births (http://names.darkgreener.com/#shakira).

Novelty precedes mimicry: 1999 was also the year of release of the popular science-fiction film *The Matrix*, the protagonist of which is named Neo, and 2000 the year marking the first appearance in ONS records of the forename Neo (http://names.darkgreener.com/#neo). This is particularly notable as the ONS dataset is a complete population sample, beginning in 1996 (note, however, that names registered to < 3 births are redacted by the ONS, so it remains possible Neo was in use prior to its fame via film). Several fads also originate in the book and television fantasy series *Game of Thrones*, particularly *Khaleesi*, a word from the fictitious Dothraki language analogous in meaning to ‘queen’; this entered UK birth records in the year the television series premiered, 2011 (as did, in 2013, *Daenerys*, the name of the khaleesi in question). Other names popularised by this series and becoming novel introductions to the ONS birth records are *Tyrion* (2011), *Sansa* (2012), *Sandor* (2013), and *Brienne* (2014). While these fads are easily related to their likely inspiration, in absolute terms, they account for a very small number of registered births.

***Expanding forename diversity***

The BMD corpus contains 143,259 unique names, of which 89,391 (62%) are registered at least once as a forename, and 48,716 (34%) only once (S5 Table). The distribution of name usage, by number of registered births, has a long tail – 29% of the total registered births (6,463,081 of 22,637,285) are represented by the top 10 most popular names (John, Mary, William, James, Thomas, Elizabeth, George, Sarah, Margaret and Joseph), 72% by the top 100, 94% by the top 500, and 97% by the top 1000.

Accordingly, the diversity of forenames (the ratio of the number of unique forenames to the total number of birth records per year) increases substantially towards the present day: by 2010 in the local BMD dataset, the forename diversity is 0.154, i.e. there are 1626 unique forenames among 10,566 records (S6 Table). There are, however, far fewer contemporary than historical birth records in the BMD dataset – from 1838-1950 there are, per year, at least tenfold as many. In general, the set of modern names is larger than the set of traditional names, due to increases both in the freedom of movement of people, and in social freedom towards following fashion rather than tradition. Consequently, in recent years, the most popular forename is not only more likely to change year-on-year but proportionately fewer people are given it.

This is especially apparent with the higher-resolution ONS dataset, which contains all registered live births in England and Wales between 1996 and 2016. Within 20 years, the number of distinct forenames, registered in at least 3 births, increases from 8297 to 13,212, with an associated increase in forename diversity (that is, ratio of unique forenames to total names registered) from 0.014 to 0.021 (S15 Table). This can be interpreted to mean that in 1996, 1.4% of all registered births were uniquely named but by 2016, 2.1%. In addition, for each year of the 20 year dataset, the majority of forenames are registered to a minority of people. Approximately 65% of the forenames are registered to fewer than 10 people - from 5463 of the 8297 forenames registered in 1996, to 8549 of the 13,212 forenames registered in 2016 (S15 Table). The proportion of names uniquely registered in a given year (that is, registered in only one year within the 20 year dataset) also increases, from approximately 3 to 5% of all unique forenames per year.

However, this proportion, while high, is unavoidably overestimated: to protect the identity of individuals, names registered to fewer than 3 births were redacted prior to making the dataset available. As a corollary of this, however, the proportion of names registered to fewer than 10 individuals a year is also underestimated.

Many of these unique names are novel coinages – that is, derivatives of existing names – rather than an outside introduction to the pool of possible choices (such as *Kylie*, discussed above). Thousands of novel names have been created simply by appending a second name to the first, registering a new, hyphenated, forename. The majority of these novel coinages are for female names. In particular, 13 second names have been registered to > 100 distinct forenames: *-Rose* (registered as a second name to 409 different forenames), *-Mae* (271), *-Leigh* (237), *-Lee* (223), *-May* (216), *-James* (181), *-Louise* (177), *-Rae* (165), *-Mai* (156), *-Marie* (150), *-Grace* (135), *-Jay* (112), and *-Ann* (111). The increasing use of these combinations (from 2.5% of the total number of unique forenames registered in 1996, to 9.1% in 2016; see Figure S1) has expanded forename diversity enormously. For instance, the name *Amelia* reached its proportionately highest usage in 2012, as the most popular female name of the year (1.05% of registered births). Within the 20 year span of the ONS dataset, however, there have also been 29 registered combinations for *Amelia* (*-Alice, -Ann, -Anne, -Brooke, -Elizabeth, -Faith, -Faye, -Grace, -Hope, -Jade, -Jane, -Jayde, -Jayne, -Jean, -Jo, -Lee, -Leigh, -Lillie, -Lilly, -Lily, -Louise, -Mae, -Mai, -Marie, -May, -Paige, -Rae, -Rose,* and *-Skye*), 7 spelling variants (*Ameliah, Amellia, Amelija, Ameliya, Ameliyah, Amelja,* and *Amelya*) and the hyphenated versions thereof (e.g., *Ameliah-Rose*), plus a similar name *Amelie* with 5 of its own variants (*Amelea*, *Ameleah*, *Ameli*, *Amellie*, *Amely*) and 4 of its own hyphenated additions (*-Grace*, *-Mae*, *-Mai*, and *-Rose*). Disregarding the similar name *Amelie*, the set of variants for *Amelia* total 3539 records: 5% of the 68,325 records of *Amelia*. Consequently, while a specific variant may be rare, in sum these variants notably reduce the number of individuals registered with popular names.

There are also numerous names with variable suffixes – that is, names which can be registered with one of multiple common endings, such as -ee, -ey, -i, -ie or -y (for example, *Hollee*, *Holley*, *Holli*, *Hollie* or *Holly*). We obtained a subset of 61 ‘root’ names in which all 5 possible endings have been used at least once in the dataset, such as *Am-* (*Amy*),  *Hayl-* (*Hayley*) and *Kat-* (*Katie*). Over the 20 year span of the dataset, 1.5% to 2% of the total number of unique forenames are accounted for by this set of variants (which represent 61x5 = 305 unique names) (Table 15). Notably, the proportion of names registered in each year increases over time, from 53% in 1996 (i.e. 160 of 305 names) to a peak of 73% in 2009 (i.e. 223 of 305 names) (S1 Figure).

This greatly increases the number of possible names, irrespective of outside introductions to the pool of options. If adding -eigh to the set of possible endings, 16 names are registered with all 6 possibilities: variants of *Lily, Harley, Holly, Emily, Lacy, Karly, Ellie, Henleigh, Tilly, Marley, Ryley, Charlie, Kelsey, Ashley, Carly* and *Keeley*. If adding -ea, 9 names are registered with all 7 possibilities: *Harley*, *Keeley*, *Ashley*, *Kelsey*, *Ryley*, *Charlie*, *Ellie*, *Marley*, *Emily* (and finally, with the addition of -eah, 2 of these names, *Ashley* and *Ellie*, have all 8 possible endings). Furthermore, 82 names have at least 4 of the 5 most common variant endings (*Sydnee*, *Sydney*, *Sydni*, and *Sydnie* are all recorded; *Sydny* is not), 181 names at least 3 (*Jenny*, *Jenni* and *Jennie* are recorded; *Jenney* and *Jennee* are not), and 340 names at least 2 (*Polly* and *Pollie* are recorded; *Polli*, *Polley* and *Pollee* are not).

**References**

1. Wilson S (1998) The Means of Naming: A Social and Cultural History of Personal Naming in Western Europe. London: UCL Press.

2. Mitterauer M (1993) Ahnen und Heilige: Namengebung in der europäischen Geschichte. Munich: Beck.

3. Scheuble LK, Johnson DR (2016) Keeping Her Surname as a Middle Name at Marriage: What Predicts this Practice Among Married Women Who Take Their Husband’s Last Name? Names 64: 202-216.
