# Supplementary figures and images for: Network analysis of the social and demographic influences on name choice within the UK (1838-2016)
Source: PLoS One. 2018 Oct 31;13(10):e0205759. doi: 10.1371/journal.pone.0205759 (PMC6209202; doi:10.1371/journal.pone.0205759)

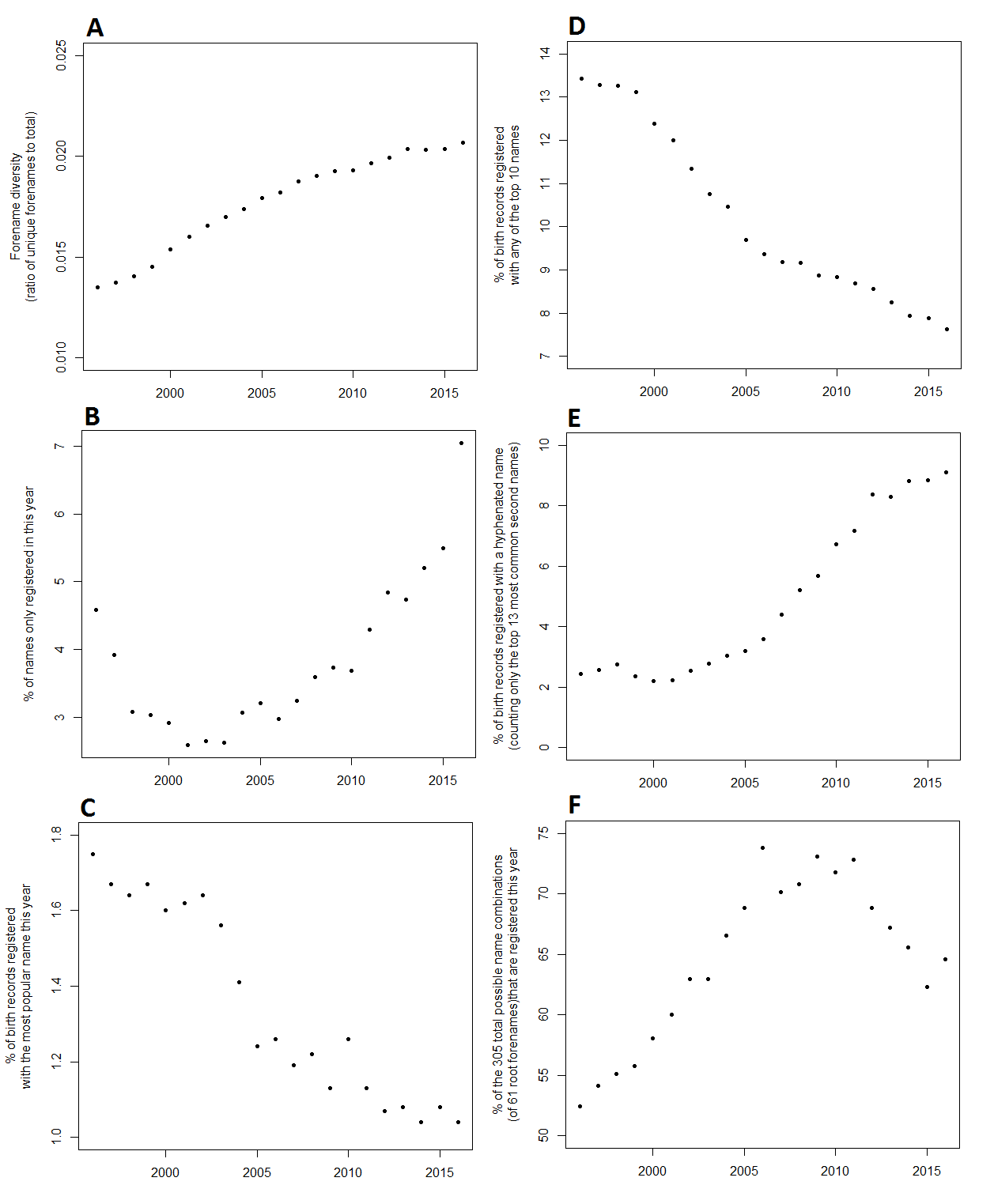

Supplement: S1 Fig — Overview of the ONS dataset, showing (A) the forename diversity per year (the number of unique forenames as a proportion of the number of births), (B) the percentage of names only registered in this year, (C) the percentage of records registered with the most popular name of that year, (D) the percentage of records registered with any of the 10 most popular names of that year, and (E) the percentage of records registered with a hyphenated forename (counting only the 13 most common second names in a hyphenated name). Graph (F) uses a subset of 61 ‘root’ forenames in which all 5 common endings (-ee, -ey, -i, -ie, or -y) have been registered at least once in the dataset. The figure shows the percentage of the total possible name combinations that are registered this year, i.e. the number of names used out of 61x5 = 305 possibilities. (TIF) [file pone.0205759.s017.tif]
